# Moving instead of asking? Performance-based tests and BASFI-questionnaire measure different aspects of physical function in ankylosing spondylitis

**DOI:** 10.1186/ar3765

**Published:** 2012-03-08

**Authors:** Salima FE van Weely, J Christiaan van Denderen, Martijn PM Steultjens, Marike van der Leeden, Michael T Nurmohamed, Joost Dekker, Ben AC Dijkmans, Irene E van der Horst-Bruinsma

**Affiliations:** 1 Reade, Centre for Rehabilitation and Rheumatology, Dr. Jan van Breemenstraat 2, PO Box 58271, 1040 HG Amsterdam, the Netherlands; 2 Glasgow Caledonian University, Institute for Applied Health Research, Glasgow City G4 0, Scotland, UK; 3 VU University Medical Centre, Department of Rehabilitation Medicine, EMGO Institute, PO Box 7057, 1007 MB Amsterdam, The Netherlands; 4 VU University Medical Centre, Department of Rheumatology, PO Box 7057, 1007 MB Amsterdam, The Netherlands; 5 VU University Medical Centre, Department of Psychiatry, PO Box 7057, 1007 MB Amsterdam, The Netherlands

## Abstract

**Introduction:**

Ankylosing Spondylitis (AS) is characterised by limitations in physical function. The Bath Ankylosing Spondylitis Functional Index (BASFI) is considered to be the gold-standard to assess physical function in AS patients. However, the BASFI questionnaire is a self-reported outcome measure and susceptible to subjective interpretation (under- or over-estimation). More objective outcome measures, like performance-based tests, could provide an objective outcome measurement for the evaluation of limitations in physical function. Therefore, the primary aim of this study was to determine the association between performance-based measures and the BASFI questionnaire.

**Methods:**

In this cross-sectional study 126 AS patients completed the BASFI questionnaire and eight performance-based tests based on BASFI-items. Each test received three scores: one for performance (time or points) and a score for exertion and pain experienced during performance (using modified Borg-scale and VAS 0-100 mm, respectively). Linear regression analyses were used to assess the associations between the BASFI questionnaire and performance-based tests.

**Results:**

The univariable association between performance and BASFI-score was moderate with a R-square of 0.31 and Beta of 0.56 (p's < 0.05). In a multivariable analysis, the association between performance, exertion and pain on the one hand and BASFI-score on the other was assessed; R-square increased to 0.54: the Beta's for exertion and pain during performance were 0.38 and 0.26, respectively; the Beta for performance decreased to 0.19 (p's < 0.05).

**Conclusions:**

This study demonstrates that alongside actual performance, patients seem to incorporate exertion and pain in their assessment of perceived physical function on the BASFI questionnaire. Performance-based tests could provide an objective outcome measurement for the evaluation of physical function and give relevant new information in addition to the BASFI questionnaire.

## Introduction

Limitations in physical function due to inflammation, pain and reduced spinal mobility are an important feature of ankylosing spondylitis (AS). Physical function is, therefore, considered an important outcome measure for evaluating the disease course and the effectiveness of interventions in AS patients [1,2]. The preferred assessment methods for evaluating limitations in physical function in AS patients are self-reported outcome measures such as the Bath Ankylosing Spondylitis Functional Index (BASFI) [3] or the Dougados Functional Index (DFI) [4]. Both indexes are disease-specific outcome parameters that have been proven to have adequate clinimetric properties and to be reflective of patients' perspectives [5-8].

The BASFI questionnaire is currently the most widely used instrument to assess physical function in AS patients. A self-reported measure such as the BASFI refers to a method in which an individual is asked to indicate his/her perceived level of function during daily activities, described in standardised questions [9]. An alternative method for assessing limitations in physical function is the use of performance-based tests. In these measures an individual has to perform one or more specific activities that are evaluated in a standardised manner, mostly time to complete the activity [9,10].

Although self-reported and performance-based measures both claim to assess the physical function domain, numerous studies have reported only moderate relations between both measurements. These moderate relations were found, among others, in elderly patients, and those with chronic low back pain and osteoarthritis [9,11-14]. It has been suggested that (time-based) performance and self-reported measures assess different aspects of the physical function domain [15-17]. Self-reported measures of limitations in physical function represent what people experience when they are performing activities rather than their true ability to be able to perform the activities [9,11,18]. This may lead to discrepancies between perceptions of the individual assessed using a self-reported measure and their true ability (underestimation or overestimation, e.g. due to personality traits, language or depression) [9-11,19]. In addition, discordance between observed and perceived physical function measured with a self-reported questionnaire has been reported in patients with AS, rheumatoid arthritis and fibromyalgia [20,21].

Recently, we developed performance-based tests of physical function for AS patients based on items described in the BASFI questionnaire [22]. The performance score (i.e. time to perform an activity or points scored), as well as exertion and pain during performance were measured. We reported that these tests showed adequate to excellent test-retest reproducibility on all test components (performance score, exertion and pain) [22]. However, until now no information has been available on how performance-based physical function relates to self-reported physical function as assessed with the BASFI questionnaire. Similarly, no information is available on how exertion and pain during performance have impact on self-reported physical function, as assessed with the BASFI questionnaire. Therefore, the primary aim of the present study was (i) to determine the association between performance-based tests and the BASFI questionnaire in AS patients, and (ii) to determine the association between exertion and pain during performance and the BASFI questionnaire. The secondary aim of this study was (iii) to determine the associations between performance-based tests of physical function and disease activity (as assessed with the Bath Ankylosing Spondylitis Disease Activity Index; BASDAI) [23] as well as impairments in mobility of the hips and spine (as assessed with the Bath Ankylosing Spondylitis Metrology Index; BASMI) [24].

## Materials and methods

Patients participating in this study were recruited from a large outpatient centre for rehabilitation and rheumatology, Reade in Amsterdam. Enrolment took place from May 2006 to May 2010. The following inclusion criteria were applied: diagnosis of AS according to the modified New York criteria [25], age of 18 years or older and sufficient command of the Dutch language. Patients with pulmonary, cardiovascular or neurological co-morbidity affecting the patient's ability to perform daily activities were excluded. The study was approved by the medical ethical committee of the Slotervaart Hospital and Reade. All patients gave written informed consent according to the Declaration of Helsinki.

### Design and measures

A cross-sectional design was applied to determine the associations between (i) the performance scores [22] and the BASFI questionnaire [3,26], (ii) the performance scores, exertion, pain and the BASFI questionnaire, and (iii) between the performance scores and BASDAI and BASMI, respectively. Patients completed the performance tests, BASFI, BASDAI and BASMI within a one-month period. It was assumed that during this period the patient's physical condition remained unchanged. The same assessor measured all subjects on the performance-based tests and was blinded for the results of the BASFI, BASDAI and BASMI.

The eight performance-based tests based on items of the BASFI included: climbing stairs (no. 1), bending (no. 2), reaching up (no 3), putting on socks (no. 4), reclining and declining from a chair (no. 5), getting up from the floor (no. 6), looking over the shoulder (no. 7) and a physically demanding activity (no. 8) as previously described by van Weely et al. [22]. In all tests the time taken to perform the task was measured in seconds, except for no. 7 (looking over the shoulder) in which the range of vision was recorded in points. Pain and exertion experienced during performance were assessed directly following each test using a 10-cm horizontal visual analogue scale (VAS) and Borg's modified scale [27], respectively. The Borg scale is a rating of perceived exertion and was developed to describe a person's perception of exertion during exercise. In this way, each performance test received three scores: a performance score (time or points) and scores for pain and exertion as experienced during performing the test.

We used the generally accepted BASDAI [23] as the outcome parameter for disease activity, because common laboratory measures of inflammation have only a limited ability to corroborate patient reported health status or clinician observed impairments. Impairments in spinal and hip mobility were assessed by the BASMI [24]. This is a combined index comprising of measurements of lateral spinal flexion, tragus-to-wall distance, lumbar flexion, intermalleolar distance and cervical rotation.

### Statistical analyses

Descriptive statistics were computed by calculating means and standard deviations (SDs) for all continuous data and percentages for categorical data. Prior to determining the various associations with the performance-based tests, the performance scores were transformed into Z-scores. The association between the separate tests (internal consistency) was established, and a total performance score was established by calculating a mean Z-score.

To assess the association between performance-based tests of physical function and BASFI questionnaire, univariable and multivariable linear regression analyses were performed. Univariable analysis was performed with performance as independent variable and the BASFI-score as dependent variable. In multivariable analyses performance as well as exertion and pain during performance were entered as independent variables and the BASFI-score as dependent variable.

Univariable regression analyses were also used to establish the associations between performance tests of physical function and disease activity (BASDAI) as well as between performance tests and impairments in mobility of the hips and spine (BASMI). All analyses were performed using SPSS for Windows 18.0 (SPSS Inc, Chicago, IL, USA). Statistical significance was set at the *P *< 0.05 level.

## Results

### Descriptive statistics

The study population consisted of 139 consecutive AS patients. BASFI data were missing for 13 patients (9.4%) and they were therefore excluded. No patients were excluded for having pulmonary, cardiovascular or neurological co-morbidity affecting the patient's ability to perform daily activities. Accordingly, 126 patients (70.6% men) with a mean age of 45.9 years were included in the analyses. Table 1 displays the baseline characteristics. Disease activity was high with a mean BASDAI (SD) of 5.2 (± 2.3). Medication was used by 80% of the study population and only 4% was treated with TNF-blockers (biologicals) at the time of the study. Patients' characteristics, means and standard deviations (SD) of the BASFI, BASDAI and BASMI are displayed in Table 1.

**Table 1:** Characteristics of 126 patients with ankylosing spondylitis

Men % (n)	70.6 (89)
Age* (years)	45.9 ± 11.5
Symptom duration* (years)	8.1 ± 7.4
Disease duration* (years)	14.7 ± 10.0
Current medication % (n)	
None	12.7 (16)
NSAIDs	61.9 (78)
Biologicals	4.0 (5)
Combination	10.3 (13)
Other^#^	4.0 (5)
HLA-B27+ % (n)	77.8 (98)
ESR* (mm/hour)	10.4 ± 18.4
Extra-spinal symptoms % (n)	
psoriasis	4.8 (6)
uveitis	19.8 (25)
inflammatory bowel disease	4.0 (5)
arthritis	38.1 (48)
BASFI* (score 0-10)	4.9 ± 2.3
BASDAI* (score 0-10)	5.2 ± 2.3
BASMI* (score 0-10)	4.1 ± 2.0

* mean ± standard deviation

^# ^e.g. sulfasalazine, methotrexate.

BASDAI, Bath Ankylosing Spondylitis Disease Activity Index; BASFI, Bath Ankylosing Spondylitis Functional Index; BASMI, Bath Ankylosing Spondylitis Metrology Index; ESR, { Erythrocyte Sedimentation Rate NSAIDs, non-steroidal antiinflammatory drugs.

### Relations between the performance scores (internal consistency)

Based on analysis for internal consistency items 1 to 6 and 8 (stair climbing, bending, reaching, putting on socks, reclining and declining from a chair, getting up from the floor and physically demanding activity) can be regarded as representations of the same domain. Item 7 (looking over the shoulder) does not belong to this domain. Inter-item correlations of items 1 to 6 and 8 with item 7 were low (0.25-0.32). Factor analysis (principal components) showed one component, with items 1 to 6 and 8 loading in the range from 0.65 to 0.85, while item 7 loaded only 0.38. Chronbach's alpha increased from 0.85 to 0.87 when item 7 was deleted. Therefore, univariable and multivariable analyses were performed separately for items 1 to 6 plus 8, and for item 7.

### Performance tests and the BASFI questionnaire

Results of the uni- and multivariable regression analyses are shown in Table 2. The univariable association between performance tests 1 to 6 plus 8 and BASFI-score was moderate with a R-square of 0.31 and Beta of 0.56. In the multivariable association R-square increased to 0.54: Beta's for exertion and pain during performance were 0.38 and 0.26, respectively, while the Beta for performance decreased to 0.19 (*P *< 0.05).

**Table 2:** Univariable and multivariable associations of performance-based tests with the BASFI questionnaire (tests 1 to 6 and 8 together, test 7 separate) (*n *= 126)

Tests 1 to 6 plus 8	R-square		Beta	*P *value
Univariable	0.31	Performance	0.56	0.000
Multivariable	0.54	Performance	0.19	0.018
		Exertion	0.38	0.000
		Pain	0.26	0.008

**Test 7**				
Univariable	0.39	Performance	0.63	0.000
Multivariable	0.51	Performance	0.49	0.000
		Exertion	0.00	0.991
		Pain	0.36	0.001

Tests: 1) climbing stairs, 2) bending, 3) reaching up, 4) putting on socks, 5) reclining and declining from a chair, 6) getting up from the floor, 7) looking over the shoulder, 8) physically demanding activity. BASFI, Bath Ankylosing Spondylitis Functional Index.

In the analyses on performance-test 7 (looking over the shoulder), similar results were seen. The univariable association was moderate with a R-square of 0.39 and Beta of 0.63. In the multivariable association R-square increased to 0.51 and the Beta for the performance test decreased to 0.49 (*P *< 0.05). The Beta for pain during performance was 0.36 (*P *< 0.05); the Beta for exertion during performance was 0.00 and not significant.

### Performance tests, BASDAI and BASMI

Table 3 shows the results of the univariable associations between performance tests and BASDAI as well as BASMI. The results indicate that performance tests of physical function are more strongly associated with impairments in spinal and hip mobility (BASMI) than with self-reported disease activity (BASDAI). The association between performance tests and BASDAI was low with a R-square of 0.08 and Beta of 0.28 for tests 1 to 6 plus 8 and a R-square of 0.00 with a Beta close to zero (-0.06; *P *= 0.534) for test 7. The association of the performance tests with the BASMI was higher with a R-square of 0.24 and Beta of 0.50 for tests 1 to 6 plus 8. For test 7 R-square was 0.60 and Beta was 0.77.

**Table 3:** Associations of performance-based tests with the BASDAI questionnaire and BASMI (tests 1 to 6 plus 8 together, test 7 separate) (*n *= 126)

Tests 1 to 6 plus 8	R-square	Beta	*P *value
BASDAI	0.08	0.28	0.002
BASMI (*n *= 98)	0.24	0.50	0.000

**Test 7**			
BASDAI	0.00	-0.06	0.534
BASMI (*n *= 97)	0.60	0.77	0.000

Tests: 1) climbing stairs, 2) bending, 3) reaching up, 4) putting on socks, 5) reclining and declining from a chair, 6) getting up from the floor, 7) looking over the shoulder, 8) physically demanding activity. BASDAI, Bath Ankylosing Spondylitis Disease Activity Index; BASMI, Bath Ankylosing Spondylitis Metrology Index.

## Discussion

The present study shows that the association between performance-based tests of physical function and the BASFI questionnaire was moderate. The BASFI questionnaire has a stronger association with exertion and pain during performance than with actual performance. Furthermore, we have shown that the performance tests are more strongly associated with impairments in spinal and hip mobility (BASMI) than with disease activity (BASDAI). This demonstrates that performance-based tests of physical function and the BASFI questionnaire do not measure the same aspects of the physical function domain in AS patients. Performance-based tests seem to measure actual physical function and the BASFI questionnaire seems to measure perceived physical function. When patients define their physical function on the BASFI questionnaire, exertion and pain experienced during performing activities seem to be of greater influence on the score than actual performance. Consequently, performance-based tests can give additional information on physical function and can provide an objective outcome measurement for the evaluation of interventions.

Performance-based measures for the evaluation of physical function in AS patients have not been reported previously. Moderate associations between performance-based and self-reported measures of physical function, such as with the BASFI questionnaire in our study, have been found consistently in other patient groups (elderly, chronic low back pain, osteoarthritis) [10,12-15]. Three explanations for the moderate correlations between the BASFI questionnaire and the performance-based tests have to be considered. Firstly, self-reported measures such as the BASFI questionnaire incorporate multiple aspects relating to physical function such as time to complete a task and exertion and pain experienced during activities, whereas performance-based tests primarily measure time to complete a task. Additionally, self-reported measures are influenced by psychological factors. Recent findings by Brionez et al. [28] support this. They found that in patients with AS helplessness, depression and passive coping accounted for significant variability in self-reported functional limitations as measured with the BASFI questionnaire. They advised that psychological health should be examined and accounted for when assessing functional status in AS patients [28]. Psychological health was not measured in our cohort of AS patients, and could therefore not be taken into account when interpreting our results. Secondly, discrepancies in the association between performance-based tests and the BASFI questionnaire may occur due to underestimation of limitations in physical function. Patients with longer disease duration may tolerate more limitations in physical function and adapt to a certain amount of declining function. Thirdly, the association between performance-based physical function and the BASFI questionnaire is influenced by the reliability of these measures [14]. We previously showed that the reliability of the performance-based measures is good [22] and equivalent with the BASFI questionnaire [3,6,8]. However, due to the lack of perfect reliability, the maximal obtainable association between performance-based measures and the BASFI questionnaire is limited.

We showed that not all performance items belong to the same domain. Performance items no. 1 to 6 and 8 (stair climbing, bending, reaching, putting on socks, reclining and declining from a chair, getting up from the floor and doing a physically demanding activity) seem to belong to one domain as opposed to looking over the shoulder. This may be due to the fact that items 1 to 6 and 8 are activities that concern complex movements by which the heart rate may increase and stamina, muscle power and coordination are necessary, whereas item 7 (looking over the shoulder) is an isolated movement of the cervical spine that refers to an impairment in mobility.

The results from this study also show that performance-based physical function is associated with impaired mobility of the spine and hip due to structural changes (as measured by the BASMI). Although related, both concepts are not equivalent. In the BASMI the range of motion in a static position is measured. Whereas, performance-based physical function is established by the timing of a daily activity comprising of complex movements. Executing an activity such as standing up from the floor will be affected by an impaired mobility of the spine and hip, but also by muscular strength, stamina and coordination. The sum of these properties seems to be measured by a performance-based test. Disease activity as measured with the BASDAI was only weakly related to performance-based physical function.

In our opinion, two study limitations warrant further consideration. Firstly, the BASFI and the performance-based tests were completed within one month, with the assumption that patients had an unchanged physical condition in this period. Variability in disease activity for example might have influenced physical condition between both measurement points. A study by Berthelot et al. in spondylarthropathy patients indicated that BASFI scores often show variations over time in a given patient [29]. However, these variations concerned self-reported physical function, and do not correspond with our previous findings regarding the reliability of performance-based physical function measures over time [22]. In addition, mean disease duration was 14.7 years and most patients were stable on medication. As changes in physical function take time to develop, it is unlikely that major changes in physical function within only a one-month period have occurred. Therefore, the influence of therapy and timing on the completion of the BASFI questionnaire and performance-based tests on our results is expected to be only marginal.

Secondly, the time of day at which the tests were performed could have influenced our results. When assessing early in the morning or late in the afternoon patients could have had more difficulty performing tests due to morning stiffness or fatigue for example. However, patients could choose the time of the assessment, therefore it is not likely that this played a big role.

For future research, additional data on the responsiveness to interventions, such as the start of TNF blockers, have to be established. Another focus for future research is to select a group of performance-based tests, generating the equivalent information as the full set, which is feasible for use in daily clinical practice. To improve the feasibility of the performance-based tests in daily clinical practice adaptations to the scoring method could be considered.

## Conclusions

To conclude, this study clearly demonstrates that associations between performance-based measures of physical function and the BASFI questionnaire are moderate. Alongside actual performance, patients seem to incorporate exertion and pain in their assessment of perceived physical function on the BASFI questionnaire. Performance-based tests could provide an objective outcome measurement for the evaluation of physical function and give relevant new information in addition to the BASFI questionnaire.

## Abbreviations

AS: ankylosing spondylitis; BASDAI: Bath Ankylosing Spondylitis Disease Activity Index; BASFI: Bath Ankylosing Spondylitis Bath Functional Index; BASMI: Bath Ankylosing Spondylitis Metrology Index; DFI: Dougados Functional Index; SD: standard deviation; TNF: tumour necrosis factor; VAS: visual analogue scale

## Competing interests

The authors declare that they have no competing interests.

## Authors' contributions

All authors have read and approved the manuscript for publication. SvW participated in the design of the study, collected all data, performed the statistical analysis and drafted the manuscript. CvD participated in the design of the study, acquisition of data and helped to draft the manuscript. MS made substantial contributions to conception, design and coordination of the study, helped with the analysis and to draft the manuscript. MvdL, MN and BD helped to draft the manuscript. JD made substantial contribution to the interpretation of data and revising it critically for import intellectual content and gave final approval of the version to be published. IvdHB made substantial contributions to conception and design of the study, interpretation of data, revising it critically for important intellectual content and gave final approval of the version to be published.
